# The effectiveness of home versus community-based weight control programmes initiated soon after breast cancer diagnosis: a randomised controlled trial

**DOI:** 10.1038/s41416-019-0522-6

**Published:** 2019-08-01

**Authors:** Michelle Harvie, Mary Pegington, Debbie McMullan, Nigel Bundred, Karen Livingstone, Anna Campbell, Jane Wolstenholme, Eleanora Lovato, Helen Campbell, Judith Adams, Sean Speed, Julie Morris, Sacha Howell, Anthony Howell

**Affiliations:** 1 Prevent Breast Cancer Research Unit, The Nightingale Centre, Manchester University NHS Foundation Trust, Manchester, UK; 2 Manchester Breast Centre, Manchester Cancer Research Centre, University of Manchester, Manchester, UK; 3 Division of Cancer Sciences, Faculty of Biology, Medicine and Health, University of Manchester, Manchester, UK; 4 The Nightingale Centre, Manchester University NHS Foundation Trust, Manchester, UK; 5 School of Applied Sciences, Edinburgh Napier University, Edinburgh, UK; 6 Nuffield Department of Population Health, Health Economics Research Centre, University of Oxford, Oxford, UK; 7 National Perinatal Epidemiology Unit, University of Oxford, Oxford, UK; 8 Clinical Radiology, Faculty of Medicine Biology and Health, University of Manchester, Manchester, UK; 9 The School of Nursing, Midwifery and Social Work, University of Manchester, Manchester, UK; 10 Department of Medical Statistics, Manchester University NHS Foundation Trust, Manchester, UK; 11 The Christie NHS Foundation Trust, Manchester, UK

**Keywords:** Oncology, Breast cancer

## Abstract

**Background:**

Breast cancer diagnosis may be a teachable moment for lifestyle behaviour change and to prevent adjuvant therapy associated weight gain. We assessed the acceptability and effectiveness of two weight control programmes initiated soon after breast cancer diagnosis to reduce weight amongst overweight or obese women and prevent gains in normal-weight women.

**Methods:**

Overweight or obese (*n* = 243) and normal weight (*n* = 166) women were randomised to a three-month unsupervised home (home), a supervised community weight control programme (community) or to standard written advice (control). Primary end points were change in weight and body fat at 12 months. Secondary end points included change in insulin, cardiovascular risk markers, quality of life and cost-effectiveness of the programmes.

**Results:**

Forty-three percent of eligible women were recruited. Both programmes reduced weight and body fat: home vs. control mean (95% CI); weight −2.3 (−3.5, −1.0) kg, body fat −1.6 (−2.6, −0.7) kg, community vs. control; weight −2.4 (−3.6, −1.1) kg, body fat −1.4 (−2.4, −0.5) kg (all *p* < 0.001). The community group increased physical activity, reduced insulin, cardiovascular disease risk markers, increased QOL and was cost-effective.

**Conclusions:**

The programmes were equally effective for weight control, but the community programme had additional benefits.

**Clinical trial registration:**

ISRCTN68576140

## Background

Observational studies indicate that excess weight at breast cancer (BC) diagnosis and significant weight gain (5–10%) thereafter are associated with increased BC specific and all-cause mortality^1–4^ greater side effects of treatment^5,6^ and decreased quality of life (QOL).^7^ Significant numbers of BC patients are overweight (30%) or obese (25%) at diagnosis^8^ and gain ≥ 5% weight thereafter (30–50%).^9^ These data suggest that weight loss in overweight/obese women and preventing weight gain in all patients could improve the outcome and wellbeing of women after a diagnosis of BC. A large number of trials amongst overweight or obese BC patients after diagnosis report that weight loss is feasible and safe^10,11^ however, nearly all of these were initiated long after completion of adjuvant treatment, and after the weight gain associated with diagnosis and treatment had occurred.^10,11^ Three small randomised studies demonstrate the feasibility and reasonable compliance of weight and exercise programmes based either on clinic visits or telephone interventions during chemotherapy soon after BC diagnosis.^12–14^

More detailed studies of body composition amongst patients with BC suggest that weight-related adverse effects are specifically associated with increased adiposity and reduced fat-free mass.^15–17^ Early initiation of weight control is an opportunity prevent deleterious weight gain, gains in fat and reduced fat-free mass which may occur during adjuvant treatment in the months after diagnosis.^18^ There is a potential teachable moment at diagnosis thus engagement in lifestyle behaviour change soon after diagnosis may be more effective than further down the line after diagnosis.^19,20^

Both home and supervised community programmes have been found to be effective for behaviour change in the post-treatment setting.^10,11^ The aim of the study reported here was to compare the acceptability and effectiveness of both types of intervention compared with a control group. We compared a 3-month home phone and mail programme versus a community programme with a control group receiving standard written advice. The interventions were initiated soon after diagnosis, either before or early into adjuvant treatment programmes. The weight control programmes aimed to limit gains in body fat to ≤ 1 kg over the year amongst normal-weight women (body mass index, BMI < 25 kg/m^2^), and achieve a gradual weight loss of ≥ 5% (i.e. a reduction in body fat of ≥ 3 kg) amongst women who were overweight or obese at diagnosis (BMI ≥ 25 kg/m^2^). Primary end points were change in weight, body fat and fat-free mass (FFM) at 12 months. Secondary end points included 12-month change in cardiovascular (CVD) markers, insulin sensitivity (homoeostatic model assessment, HOMA), QOL, fitness, and the relative cost-effectiveness of the programmes. We also wished to assess the generalisability of the programme and thus women were recruited from nine breast units across Greater Manchester and Cheshire.

## Methods

### Study design

A multicentre randomised controlled three-arm trial (1:1:1) within nine breast units in the Greater Manchester and Cheshire Cancer Research Network, UK, coordinated by Manchester University NHS Foundation Trust at Wythenshawe Hospital.

### Patient population

Participants were recruited within 12 weeks of surgery for invasive or in-situ primary BC. There were no age, weight or treatment restrictions since we were assessing the general applicability of the interventions in all patients diagnosed with early BC. Women were excluded if they had major physical/psychiatric conditions which would limit compliance to a diet and physical activity (PA) programme, diabetes requiring insulin or regularly taking medication known to affect body composition, e.g. daily glucocorticoids or were treated with neoadjuvant chemotherapy or endocrine therapy. Women were made aware of the trial before surgery or at their initial post-surgery appointment by their breast surgeon or research nurse.

### Randomisation and stratification

Randomisation was undertaken in the main recruiting breast unit at Wythenshawe Hospital using a minimisation programme by the trial administrator and was stratified by; chemotherapy or no chemotherapy, BMI ≥ or < 27 kg/m^2^, axillary node clearance (ANC) versus no ANC, and breast unit.

### Study interventions

#### Standard written advice (‘control’)

This group received a comprehensive booklet which explained the importance of weight control (i.e. ≥ 5% weight loss in overweight/obese and prevention of weight gain in normal-weight subjects) and physical activity (PA) after diagnosis for overall health and wellbeing, and the possible effects on BC outcome. It recommended a healthy Mediterranean type diet (45% energy from low glycaemic index carbohydrates, 30% from fat, 15% monounsaturated, 7% from saturated, 8% from polyunsaturated fat, 25% from lean protein foods, 5–7 portions fruit and vegetables/day) as described previously,^21,22^ at least 150 min/week of moderate intensity aerobic PA, two sessions of resistance PA per week and arm mobility exercise in accordance with national guidelines,^23^ and standard advice for dealing with gastrointestinal and fatigue side effects for women receiving chemotherapy.

#### Home-based phone and mail programme (‘home’)

This group received the written advice described above and individualised diet and PA advice from one of the trial dietitians and the physical activity specialist mainly by telephone after an initial face to face consultation. Diet advice included individualised food portion lists to follow a Mediterranean diet to meet estimated energy requirements for weight maintenance or an energy restriction 25% below estimated energy requirements for weight loss as described previously.^22^ Physical activity advice promoted a gradual increase towards the above targets for aerobic, resistance and arm mobility exercises which were tailored to the individual. Women were asked to estimate and report the intensity of PA using the rate of perceived exertion scale.^24^ Initial advice was given face to face in the main recruiting breast unit at Wythenshawe Hospital. The intensive 12 weeks of the programme included six fortnightly 20-min phone calls from their allocated trial dietitian to check compliance to diet and PA targets and address individual problems. This was followed by a mailed summary of goals and recommendations discussed. Women also received six fortnightly mailings which covered the same issues as the community programme. These were received on the weeks between the calls to maintain weekly contact throughout the 12-week programme (Supplementary Table 1).

#### Supervised community-based group programme (‘community’)

This group received identical written and face to face advice as the ‘home’ group, but were also asked to attend 12 weekly PA and dietary education sessions in one of five different community locations across Greater Manchester. Each session included 30 min of moderate intensity aerobic PA and 10 min of resistance and flexibility PA, followed by a 30-min diet and behaviour change education session (Supplementary Table 1). Women were monitored throughout the class to ensure that they were exercising at a moderate level (50–80% age-adjusted heart rate maximum by pulse checks and rating of perceived exertion). In addition, women were asked to undertake four aerobic and one resistance PA sessions/week at home to meet their weekly goals.

The home and community programmes used established behavioural techniques, i.e. goal setting, self-monitoring of weight and waist (weekly), diet (6 monthly food diaries), PA (daily pedometer), stress and time management, relapse prevention, and overcoming barriers.^25^ Both groups received booster phone calls from their allocated dietitian to reinforce advice, problem solve and monitor compliance at 4, 6 and 9 months. All study participants including the control group received a three month trial newsletter to encourage retention to the trial.

### Outcome measures

Trial assessments were conducted in the main recruiting breast unit at Wythenshawe Hospital at baseline, 6 and 12 months. Body weight, height, waist and hip circumference, blood pressure, fasting insulin, glucose, HOMA, total, LDL and HDL cholesterol and triglycerides were assessed and estimated using standard methods as described previously.^21,22,26^ Body fat, FFM (body mass excluding fat mass and bone mineral content) and trunk fat were determined from supine dual energy X-ray absorptiometry (DXA) scans (Hologic Discovery A with Hologic APEX software). Data from the head were excluded from all DXA measures due to the high proportion of bone mineral content known to affect the accuracy of soft‐tissue measures. Unilateral artefacts, i.e. metallic joint replacements, breast implants and lymphoedema were adjusted for by replacing the corresponding contralateral value. Physical/functional capacity was assessed from a 12 min treadmill walking test.^27^ Quality of life was assessed with the Functional Assessment of Cancer Therapy (FACT) physical wellbeing (PWB), functional wellbeing (FWB), BC specific (BCS), endocrine (ESS) and fatigue (FSS) sub scales reported as the trial outcome indicator (TOI) summary scores, e.g. TOI breast cancer (TOI-BC) = PWB + FWB + BCS; TOI endocrine symptoms (TOI-ES) = PWB + FWB + ESS; TOI fatigue (TOI-F) = PWB + FWB + FSS.^28^

### Adherence at 6 and 12 months

Dietary adherence at 6 and 12 months was assessed from seven-day food diaries in all women and analysed using WISP version 3 (Tinuviel Software, Anglesey, Wales) and levels of moderate and vigorous PA were assessed from the Scottish Physical Activity Questionnaire.^29^

### Economic evaluation

Patient-specific costs were estimated for the three trial arms from patient self-reported health care resource use diaries completed every 3 months and hospital records (hospitalisations, medication, outpatient visits, GP services used, etc) up to 12-months post-randomisation. These data were combined with EQ-5D-3L tariffs collected at baseline, 3, 6 and 12 months to estimate cost-utility.^30^

The three interventions were compared in terms of their mean total costs and Quality Adjusted Life Years (QALYs) and the incremental cost-effectiveness ratios (ICER) were estimated. This describes the incremental change in costs divided by the incremental change in health outcome. The ICER is compared against the cost-effectiveness threshold. This threshold reflects the maximum amount society is willing to pay for an additional unit of health gain. In the UK, the cost-effectiveness threshold lies between £20,000 and £30,000 per QALY.^31^

### Statistics

The sample size of 131 subjects/group was chosen to detect a 3 kg difference in change in body fat measured with DXA (assuming a common SD of 7.6 kg) between the three groups at 12 months with a two-sided significance level of 2% to adjust for multiple testing. The primary analysis was an intention to treat comparison of body fat and weight between the three groups defined at randomisation. Secondary pre-defined analyses compared body fat and weight at 6 and 12 months in the three groups stratified by whether subjects were normal weight or overweight/obese at baseline and receiving or not receiving adjuvant chemotherapy. We also assessed changes in secondary end points (insulin, HOMA, CVD risk markers, QOL, fitness) between the groups.

Outcomes at 6 and 12 months were analysed using analyses of variance regression models (ANCOVA) incorporating baseline measures as covariates. Specific pairwise comparisons between groups were carried out using Scheffe’s multiple comparison tests. The last observation carried forward (LOCF) method was used for missing outcome data. This is a conservative estimate of the ‘non-random’ missing data, as nonattendance at clinic appointments is considered more likely for those who gained weight.

## Results

Four hundred and nine women were randomised between August 2008 and February 2011, representing 42% of eligible patients from Wythenshawe Hospital which supplied complete recruitment data as reported previously.^32^ Women were randomised on average 55 (IQR 39–68) days from the date of their breast surgery.

**Fig. 1:**
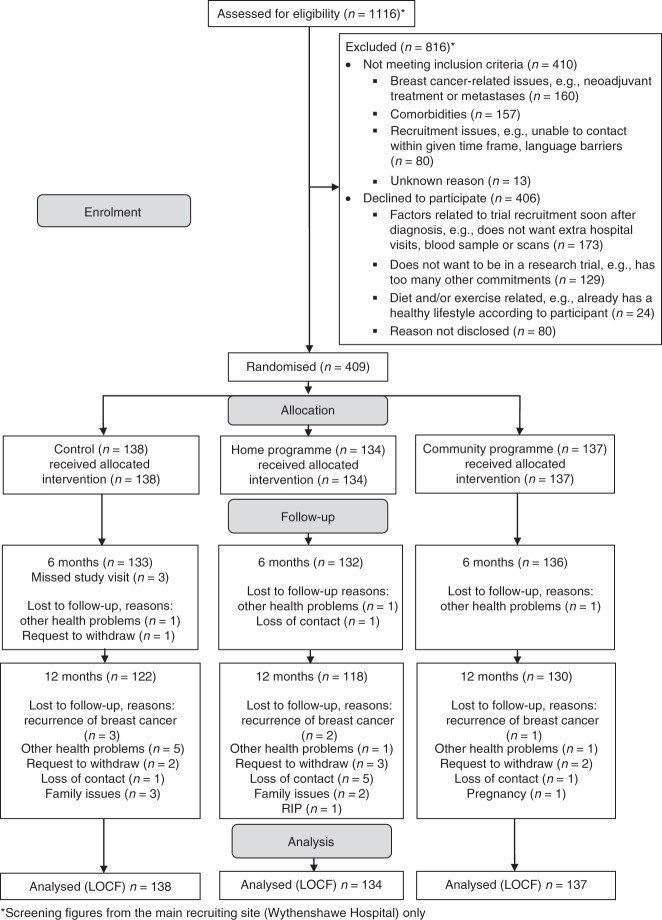
Consolidated Standards of Reporting Trials (CONSORT) flow diagram of patients recruited to the B-AHEAD trial. *Screening figures are from the main recruiting site (Wythenshawe Hospital) only

Sixteen of the control (11.5%), 16 of the home (12%) and 7 of the community group (5%) withdrew from the study due to recurrence of breast cancer (*n* = 6), other health problems (*n* = 10), family issues (*n* = 5), pregnancy (*n* = 1), request to withdraw (*n* = 8), patient had died (*n* = 1) and loss of contact (*n* = 8) (Fig. 1). At baseline, the three groups were comparable for age, ethnicity, BMI, menopausal status, tumour characteristics, breast surgery, BC treatments and prevalence of co-morbidities, and index of multiple deprivation (Table 1). Fifty-nine percent of the overall cohort were overweight or obese and 41% normal weight. Thirty-eight percent received adjuvant chemotherapy (56% overweight/obese and 44% normal weight). Women who withdrew were of comparable BMI to women who remained in the study, mean (SD) 27.3 (5.5) vs. 27.2 (5.4) kg/m^2^ *P* = 0.923, but were significantly younger, 51 (9.0) vs. 55 (10.4) years (*P* = 0.019) and more likely to be receiving chemotherapy, 13.5% of the chemotherapy group vs. 7.1% of the no chemotherapy group (*P* = 0.025).

**Table 1:** Baseline characteristics of patients randomised to the three groups

	Control	Home	Community
	(*n* = 138)	(*n* = 134)	(*n* = 137)
Age (years)	55.3 (10.5)	54.6 (11.2)	54.0 (9.2)
BMI (kg/m²)	27.6 (6.1)	26.9 (4.8)	27.0 (5.1)
*BMI Category*			
Normal weight (18.5–24.99 kg/m^2^)	58 (42.0%)	50 (37.3%)	55 (40.1%)
Overweight (≥ 25–29.99 kg/m^2^)	38 (27.5%)	56 (41.8%)	49 (35.8%)
Obese (≥ 30 kg/m^2^)	42 (30.4%)	28 (20.9%)	33 (24.1%)
Pre/peri-menopausal	56 (40.6%)	55 (41.0%)	49 (35.8%)
Post-menopausal	82 (59.4%)	79 (59.0%)	88 (64.2%)
Days between final breast surgery and randomisation	50 (39–68)	55 (36–66)	54 (40–68)
Current smoker	15 (10.9%)	17 (12.7%)	9 (6.6%)
*Ethnicity*			
White	132 (95.7%)	126 (94.0%)	129 (94.2%)
Black	2 (1.4%)	4 (3.0%)	4 (2.9%)
Asian	3 (2.2%)	4 (3.0%)	3 (2.2%)
Mixed	1 (0.7%)	0 (0.0%)	1 (0.7%)
*Social circumstances*			
Married or cohabiting	106 (76.8%)	94 (70.0%)	102 (74.5%)
Educated to degree level or higher	64 (46%)	59 (44%)	67 (49%)
*Index of multiple deprivation*			
Greater Manchester Quintile			
1 (least deprived)	66 (47.8%)	46 (34.3%)	48 (35.0%)
2	28 (20.3%)	31 (23.1%)	31 (22.6%)
3	15 (10.9%)	26 (19.4%)	24 (17.5%)
4	12 (8.7%)	15 (11.2%)	17 (12.4%)
5 (most deprived)	17 (12.3%)	16 (11.9%)	17 (12.4%)
*Co-morbidities*			
Respiratory, e.g. asthma, COPD	19 (13.8%)	15 (11.2%)	15 (10.9%)
Psychiatric, e.g. anxiety, depression	14 (10.1%)	16 (11.9%)	19 (13.9%)
Cardiovascular disease	12 (8.6%)	10 (7.4%)	11 (8.0%)
Arthritis, back or joint problems	41 (29.0%)	32 (24.0%)	29 (21.6%)
Type 2 diabetes	3 (2.2%)	7 (5.2%)	4 (2.9%)
Previous breast cancer	8 (5.8%)	7 (5.2%)	3 (2.2%)
*Tumour characteristics*			
DCIS/LCIS	13 (9.4%)	18 (13.4%)	18 (13.2%)
Invasive tumour Grade 1	25 (18.0%)	23 (17.2%)	21 (15.3%)
Invasive tumour Grade 2	61 (44.2%)	56 (41.8%)	62 (45.3%)
Invasive tumour Grade 3	40 (28.9%)	38 (28.4%)	34 (24.8%)
*Surgery*			
Mastectomy	45 (32.6%)	47 (35.1%)	48 (35.0%)
Axillary node clearance (ANC)	34 (24.6%)	34 (25.4%)	33 (24.1%)
Screen detected breast cancer	52 (51.0%)	53 (54.6%)	61 (60.4%)
*Adjuvant treatment* ^a^			
Chemotherapy	52 (37.7%)	52 (38.8%)	51 (37.2%)
Anthracycline only	29 (21.0%)	33 (24.6%)	27 (20.0%)
Anthracyline & taxane	23 (16.7%)	19 (14.2%)	24 (17.5%)
Radiotherapy	98 (71.0%)	91 (67.9%)	104 (75.9%)
Herceptin	19 (13.8%)	13 (9.7%)	11 (8.0%)
Tamoxifen	68 (49.3%)	75 (56.0%)	78 (56.9%)
Aromatase inhibitor	36 (26.1%)	34 (25.4%)	34 (24.8%)
No adjuvant treatment (no chemothetherapy, radiotherapy or other endocrine treatment)	5 (3.6%)	10 (7.5%)	8 (5.8%)

*BMI*  body mass index, *COPD* chronic obstructive pulmonary disease, *DCIS* ductal carcinoma in situ, *LCIS* lobular carcinoma in situ

Mean (SD) *n* (%) median (interquartile range)

^a^Patients were recruited between September 2008 and November 2010

### Participation in the home and community group programmes

During the initial 12-week phase of the programmes, women in the home group received mean (interquartile range) 85 (83–100) % of their six scheduled home phone calls and were sent 100% of the mailings, whilst women in the community group attended 64 (50–75)% of the 12 scheduled weekly group classes. Four- and nine-month booster calls were received respectively by 84 and 80% of the home and 83 and 82% of the community groups.

### Primary end points: change in weight and body composition

DXA data were analysed from 389 participants; 4 had no DXA scan and 16 had their DXA data omitted as they had bilateral high-density artefacts. At 12 months the home and community groups both significantly reduced weight and body fat whilst these increased in the control group. Weight reduction in the home group compared with controls was mean (95% confidence interval) −2.3 (−3.5, −1.0) kg, and body fat reduction was −1.6 (−2.6, −0.7) kg (Table 2). Weight reduction in the community group compared with controls was −2.4 (−3.6, −1.1) kg, and body fat reduction was −1.4 (−2.4, −0.5) kg (all *p* < 0.001). There were small but statistically significant reductions in FFM in the home and community groups and a modest increase in the control group. The control group experienced gains in weight and body fat between 6 and 12 months, whilst the home and community groups respectively maintained or had further reductions of weight in this period which was 3–9 months after the initial intensive 12-week programme (Table 2).

**Table 2:** Changes in weight and body composition at six and twelve months for the overall cohort

	Change over time^a^				Group difference^b^		
		Control	Home	Community	Home vs Control	Community vs Control	Community vs Home
**Weight (kg)**	Baseline	(*n* = 138) 72.5 (16.1)	(*n* = 134) 71.0 (13.9)	(*n* = 137) 71.9 (13.5)			
	Change at 6 months	0.3 (−0.4, 1.0)	−1.4 (−2.1, −0.7)	−1.1 (−1.8, −0.5)	−1.7 (−2.8, −0.6) *p* = 0.001	−1.4 (−2.6, −0.3) *p* = 0.008	0.3 (−0.9, 1.4) *p* = 1.000
	Change at 12 months	0.8 (0.1, 1.50)	−1.5 (−2.2, −0.8)	−1.6 (−2.3, −0.9)	−2.3 (−3.5, −1.0) *p* < 0.001	−2.4 (−3.6, −1.1) *p* < 0.001	−0.1 (−1.3, 1.2) *p* = 1.000
**DXA Body fat (kg)**	Baseline	(*n* = 134) 27.9 (10.1)	(*n* = 128) 27.3 (8.1)	(*n* = 127) 27.7 (8.5)			
	Change at 6 months	0.1 (−0.4, 0.5)	−1.2 (−1.7, −0.7)	−0.9 (−1.3, −0.4)	−1.3 (−2.0, −0.5) *p* = 0.001	−0.9 (−1.7, −0.1) *p* = 0.016	0.3 (−0.5, 1.1) *p* = 0.980
	Change at 12 months	0.5 (−0.7, 1.0)	−1.2 (−1.7, −0.6)	−0.9 (−1.5, −0.4)	−1.6 (−2.6, −0.7) *p* < 0.001	−1.4 (−2.4, −0.5) *p* = 0.001	0.2 (−0.8, 1.2) *p* = 1.000
**DXA Fat free mass (kg)**	Baseline	(*n* = 134) 39.1 (6.3)	(*n* = 128) 38.5 (0.6)	(*n* = 127) 39.2 (5.9)			
	Change at 6 months	0.3 (0.0, 0.5)	−0.1 (−0.4, 0.2)	−0.2 (−0.4, 0.1)	−0.4 (−0.9, 0.1) *p* = 0.200	−0.4(−0.9, 0.1) *p* = 0.140	0.0 (−0.5, 0.5) *p* = 1.000
	Change at 12 months	0.4 (0.1, 0. 7)	−0.3 (−0.6, 0.0)	−0.3 (−0.6, 0.0)	−0.7 (−1.2, −0.2) *p* = 0.005	−0.7 (−1.2, −0.1) *p* = 0.008	0.0 (−0.5, 0.6) *p* = 1.000

Mean (SD)

^a^ANCOVA, Mean (95% CI)

^b^ANCOVA with Bonferroni adjustment, Mean (95% CI)

**Fig. 2:**
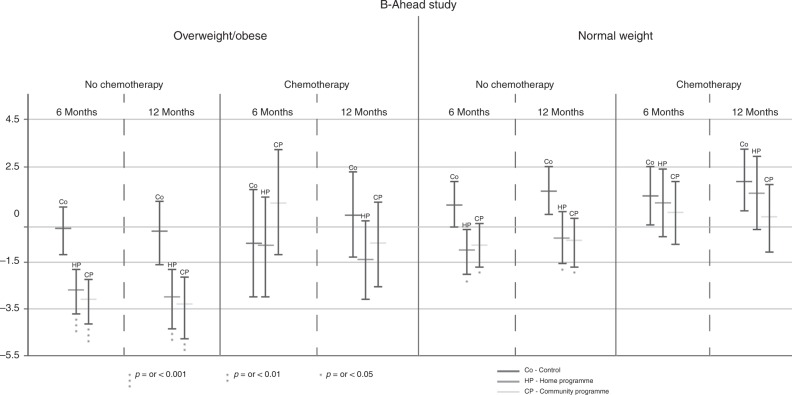
Changes in weight in overweight/ obese, normal weight and chemotherapy sub groups shown as mean 95% confidence intervals)

Pre-specified subgroup analyses indicated that weight and body composition results differed according to BMI category and whether patients were treated with chemotherapy (Table 3, Fig. 2). Both programmes induced weight loss amongst overweight/obese women who were not receiving chemotherapy, whilst weight was maintained in controls. However, the programmes did not induce weight loss amongst overweight/obese patients who were receiving chemotherapy for whom weight was maintained.

**Table 3:** Changes in weight and body composition for normal and overweight and chemotherapy sub-groups

		Change over time^a^			Group difference^b^		
		Control	Home	Community	Home vs. control	Community vs. control	Community vs. home
*Weight*							
BMI ≥ 25 kg/m^2^	Baseline	(*n* = 51) 83.0 (16.0)	(*n* = 52) 78.6 (12.0)	(*n* = 53) 78.7 (12.5)			
No chemotherapy	Change at 6 months	−0.1 (−1.1, 0.8)	−2.7 (−3.7, −1.8)	−3.1 (−4.1, −2.2)	−2.6 (−4.2, −1.0) *p* = 0.001	−3.0(−4.6, −1.4) *p* = 0.001	−0.4 (−2.0, 1.2) *p* = 1.000
	Change at 12 months	−0.2 (−1.6, 1.0)	−3.0 (−4.3, −1.8)	−3.3 (−4.7, −2.1)	−2.8 (−5.0, −0.5) *p* = 0.009	−3.1 (−5.4, −0.9) *p* = 0.003	0.3 (−2.6, 1.9) *p* = 1.000
BMI ≥ 25 kg/m2	Baseline	(*n* = 27) 79.5 (13.4)	(*n* = 32) 77.2 (11.3)	(*n* = 28) 80.7 (8.9)			
Chemotherapy	Change at 6 months	−0.7 (−2.9, 1.5)	−0.8 (−2.9, 1.0)	1.0 (−1.2, 3.2)	−0.1 (−3.8, 3.6) *p* = 1.000	1.7 (−2.1, 5.5) p = 0.840	1.8 (−1.9, 5.5) *p* = 0.700
	Change at 12 months	0.5 (−1.3, 2.3)	−1.4 (−3.1, 0.2)	−0.7 (−2.5, 1.0)	−1.9 (−4.9, 1.1) *p* = 0.370	−1.2 (−4.3, 1.9) *p* =*p* = 0.990	−0.7 (−2.3, 3.7) *p* = 1.000
BMI < 25 kg/m^**2**^	Baseline	(*n* = 35) 59.5 (4.7)	(*n* = 30) 60.2 (8.1)	(*n* = 33) 60.6 (9.3)			
No chemotherapy	Change at 6 months	0.9 (0.0, 1.7)	−1.0 (−2.0, −0.1)	−0.8 (−1.7, 0.1)	−1.9 (−3.5, −0.3) *p* = 0.011	−1.7 (−3.3, −0.2) *p* = 0.023	0.2 (−1.4, 1.8) *p* = 1.000
	Change at 12 months	1.5 (0.5, 2.5)	−0.5 (−1.6, 0.6)	−0.6 (−1.7, 0.4)	−2.0 (−3.9, −0.2) *p* = 0.028	−2.1 (−4.0, −0.3) *p* = 0.015	0.1 (−2.0, 1.8) *p* = 1.000
BMI < 25 kg/m^2^	Baseline	(*n* = 25) 62.0 (8.2)	(*n* = 20) 57.4 (7.2)	(*n* = 23) 62.0 (5.7)			
Chemotherapy	Change at 6 months	1.3 (0.1, 2.5)	1.0 (−0.4, 2.4)	0.6 (−0.7, 1.8)	−0.3 (−2.6, 2.0) *p* = 1.000	−0.7 (−2.9, 1.4) *p* = 1.000	0.4 (−2.8, 1.9) *p* = 1.000
	Change at 12 months	1.9 (0.6, 3.2)	1.4 (−0.1, 0.6)	0.4 (−0.4, 2.4)	−0.5 (−3.0, 2.0) *p* = 1.000	−1.5 (− 3.8, 2.0) *p* = 0.340	−1.0 (−3.5, 1.5) *p* = 0.980
*Body fat*							
BMI ≥ 25 kg/m^2^	Baseline	34.8 (9.4) (*n* = 49)	32.6 (6.3) (*n* = 50)	32.4 (8.2) (*n* = 49)			
No chemotherapy	Change at 6 months	−0.1 (−0.1, 0.6)	−2.3 (−3.0, −1.6)	−1.9 (−2.5, −1.2)	−2.2 (−3.4, −1.0) *p* < 0.001	−1.7 (−3.0, −0.5) *p* = 0.002	0.4 (−0.8, 1.6) *p* = 1.000
	Change at 12 months	−0.3 (−1.3, 0.6)	−2.2 (−3.1, −1.3)	−2.1 (−3.1, −1.2)	−1.9 (−3.5, −0.21) *p* = 0.022	−1.8 (−3.4, −0.1) *p* = 0.033	0.09(−1.6, 1.7) *p* = 1.000
BMI ≥ 25 kg/m^2^	Baseline	32.5 (8.7) (*n* = 27)	30.3 (6.1) (*n* = 31)	31.8 (5.6) (*n* = 27)			
Chemotherapy	Change at 6 months	−0.9 (−2.3, 0.3) (*n* = 49)	−0.7 (−1.9, 0.6) (*n* = 50)	−0.1 (−1.4, 1.3) (*n* = 49)	0.3 (−1.9, 2.5) *p* = 1.000	0.9 (−1.4, 3.2) *p* = 1.000	0.6 (−1.6, 2.8) *p* = 1.000
	Change at 12 months	0.1 (−1.4, 1.6)	−1.3 (−2.7, 0.1)	0.1 (−1.4, 1.6)	−1.4 (−3.9, 1.1) *p* = 0.530	−0.(−2.7, 2.5) *p* = 1.000	1.4 (−1.2, 3.8) *p* = 0.590
BMI < 25 kg/m^2^	Baseline	19.6 (3.5) (*n* = 33)	20.0 (5.0) (*n* = 28)	21.8 (5.5) (*n* = 30)			
No chemotherapy	Change at 6 months	0.6 (0.0, 1.3) (*n* = 25)	−1.3 (−2.0, −0.6) (*n* = 19)	−0.9(−1.6, −0.2) (*n* = 21)	−1.9 (−3.1, −0.7) *p* = 0.001	−1.5 (−2.7, 0.3) *p* = 0.010	0.4 (−0.8, 1.7) *p* = 1.000
	Change at 12 months	1.3 (0.5, 2.1)	−0.8 (−1.7, 0.0)	−0.9 (−1.7, 0.0)	−2.1 (−3.6, −0.7) *p* = 0.002	−2.1 (−3.6, −0.7) *p* = 0.002	0.0 (−1.5, 1.5) *p* = 1.000
BMI < 25 kg/m^2^	Baseline	20.0 (3.6)	19.2 (3.3)	19.9 (4.2)			
Chemotherapy	Change at 6 months	0.6 (−0.4, 1.6)	0.9 (−0.3, 2.0)	0.7 (−0.4, 1.7)	0.3 (−1.6, 2.1) *p* = 1.000	0.1 (−1.7, 1.9) *p* = 1.000	−0.2 (−2.1, 1.8) p = 1.000
	Change at 12 months	1.3 (0.2, 2.3)	1.3 (0.0, 2.5)	0.7 (−0.5, 1.9)	0.03 (−2.1, 2.0) *p* = 1.000	−0.6 (−2.6, 1.4) *p* = 1.000	−0.5 (−2.7, 1.6) *p* = 1.000
*Fat free mass*							
BMI ≥ 25 kg/m^2^	Baseline	42.0 (6.4) (*n* = 49)	41.0 (6.3) (*n* = 50)	40.3 (5.7) (*n* = 49)			
No chemotherapy	Change at 6 months	0.2 (−0.3, 5.8)	−0.3 (−0.7, 0.1)	−0.8 (−1.2, 0.4)	−0.5 (−1.2, 0.2) *p* = 0.280	−0.9 (−1.6, −0.3) *p* = 0.005	−0.4 (−1.2, 0.3) *p* = 0.370
	Change at 12 months	0.4 (−0.2, 0.9)	−0.5 (−1.1, 0.0)	−0.6 (−1.2, −0.1)	−0.9 (−1.8, 0.1) *p* = 0.072	−1.0 (−2.0, 0.1) *p* = 0.033	−0.1 (−1.1, 0.8) *p* = 1.0
BMI ≥ 25 kg/m^2^	Baseline	41.6 (6.1) (*n* = 27)	41 (6.2) (*n* = 31)	43 (4.8) (*n* = 27)			
Chemotherapy	Change at 6 months	0.2 (−0.7,1.0)	−0.8 (−0.9, 7.2)	0.1 (−0.8, 1.0)	−0.2 (−1.7, 1.2) *p* = 1.000	−0.1 (−1.2, 1.4) *p* = 1.000	0.2 (−1.3, 1.6) *p* = 1.000
	Change at 12 months	0.3 (−0.4, 1.0)	−0.5 (−1.2, 0.2)	−0.7 (−1.5, −0.1)	−0.8 (−2.0, 0.4) *p* = 0.310	−1.1 (−2.3, 0.2) *p* = 0.130	−0.3 (−1.5, 1.0) *p* = 1.000
BMI < 25 kg/m^2^	Baseline	34.3 (3.4) (*n* = 49)	35.6 (4.4) (*n* = 50)	35.4 (5.7) (*n* = 49)			
No chemotherapy	Change at 6 months	0.2 (−0.3, 0.6)	0.2 (−0.3, 0.7)	0.4 (−0.1, 0.9)	0.0 (−0.8, 0.8) *p* = 1.000	0.2 (−5.9, 1.1) *p* = 1.000	0.2 (−0.6, 1.1) *p* = 1.000
	Change at 12 months	0.4 (−1.8, 0.9)	0.1 (−0.5, 0.6)	0.6 (0.1, 1.2)	−0.3 (−1.2, 0.7) *p* = 1.000	0.3 (−0.7, 1.2) *p* = 1.000	0.6 (−0.4, 1.6) *p* = 0.540
BMI < 25 kg/m^2^	Baseline	37.1 (4.8) (*n* = 25)	33.4 (4.7) (*n* = 19)	37.0 (4.0) (*n* = 21)			
Chemotherapy	Change at 6 months	0.6 (−0.05, 1.1)	0.0 (−0.7, 0.7)	0.0 (−0.7, 0.6)	−0.6 (−1.7, 0.6) *p* = 0.750	−0.6 (−1.6, 0.5) *p* = 0.590	−0.01 (−1.2, 1.2) *p* = 1.000
	Change at 12 months	0.4 (−0.2, 1.0)	0.0 (−0.7, 0.7)	−0.1 (−0.7, 0.5)	−0.4 (−1.5, 0.7) *p* = 1.000	−0.5 (−1.5, 0.5) *p* = 0.660	−0.1 (−1.3, 1.0) *p* = 1.000

Mean (SD)

^a^ ANCOVA, m ean (95% CI)

^b^ ANCOVA with Bonferroni adjustment, m ean (95% CI)

Both programmes prevented weight gain amongst normal-weight women who were not receiving chemotherapy, whilst weight increased in controls. In contrast, the programmes did not prevent weight gain amongst normal-weight women receiving chemotherapy. In this normal weight group, patients receiving chemotherapy gained comparable amounts of weight to the non-chemotherapy patients at 12 months (Table 3, Fig. 2).

Changes in body fat were comparable to changes in weight in these subgroups. Overweight/obese non-chemotherapy patients experienced small reductions in FFM alongside weight loss, whilst FFM was maintained in the overweight/obese chemotherapy group and the chemotherapy and non– chemotherapy normal weight sub groups (Table 3).

### Secondary end points

*Biochemistry*: The community and home groups were similar with respect to weight control but significant reductions in total and LDL cholesterol, systolic blood pressure, serum insulin, HOMA, and triglycerides at 12 months were only detected in the community group (Table 4). The home group had smaller reductions in these parameters which were not significantly different from controls. Distance walked on the 12-minute walk test (a measure of fitness) increased above baseline in all groups. At 6 months the community group had significantly greater increases compared to the controls and home groups. The difference between the groups was attenuated at 12 months.

**Table 4:** Changes in secondary end points over 12 months for the overall cohort

		Change over time^a^			Group difference^b^		
		Control	Home	Community	Home vs.	Community vs.	Community vs.
					Control	Control	Home
Waist (cm)	Baseline	(*n* = 138) 95.6 (15.6)	(*n* = 134) 94.2 (13.3)	(*n* = 137) 94.7 (12.9)			
	Change at 6 months	0.1 (−0.6, 0.8)	−1.5 (−2.2, −0.8)	−2.6 (−3.3, −1.9)	−1.6 (−2.8, −0.4) *p* = 0.006	−2.7 (−3.9, −1.4) *p* < 0.001	−1.1 (−2.3,0.2) *p* = 0.110
	Change at 12 months	0.4 (−0.5, 1.2)	−1.7 (−2.6, −0.8)	−2.1 (−3.0, −1.3)	−2.1 (−3.6, −0.5) *p* = 0.003	−1.7 (−2.6, −0.8) *p* < 0.001	−0.5 (−2.0, 1.0) *p* = 1.000
Hip (cm)	Baseline	(*n* = 138) 104.5 (11.2)	(*n* = 134) 102.3 (8.6)	(*n* = 137) 103.7 (9.8)			
	Change at 6 months	0.3 (−0.4, 0.9)	−1.2 (−1.8, −0.5)	−1.6 (−2.2, −1.0)	−1.4 (−2.6, −0.3) *p* = 0.006	−1.8 (−3.0, −0.7) *p* < 0.001	−0.4 (−1.5, 0.7) *p* = 1.000
	Change at 12 months	0.9 (0.2, 1.6)	−1.1 (−1.8, −0.4)	−1.4 (−2.1, −0.7)	−2.0 (−3.2, −0.8) *p* < 0.001	−2.4 (−3.6, −1.1) *p* < 0.001	−0.4 (−1.6, 0.9) *p* = 1.000
DXA trunk fat (kg)	Baseline	(*n* = 134) 13.1 (5.3)	(*n* = 129) 12.9 (4.7)	(*n* = 128) 13.2 (4.7)			
	Change at 6 months	0.0 (−0.3, 0.2)	−0.7 (−0.9, −0.4)	−0.5 (−0.75, −0.25)	−0.7 (−1.1, −0.2) *p* = 0.001	−0.5 (−0.9, 0.0) *p* = 0.019	0.2 (−0.3, 0.6) *p* = 1.000
	Change at 12 months	0.2 (0.0, 0.5)	−0.7 (−1.0, −0.4)	−0.5 (−0.9, −0.3)	−0.9 (−1.4, −0.4) *p* < 0.001	−0.8 (−1.3, −0.3) *p* = 0.001	0.2 (−0.4, 0.7) *p* = 1.000
Glucose^c^ (mmol/L)	Baseline ^d^	(*n* = 134) 5.1 (3.2, 7.6)	(*n* = 124) 5.2 (4.1, 16.0)	(*n* = 134) 4.93 (4.0, 6.3)			
	Ratio of change at 6 months	1.008 (0.994, 1.021)	0.994 (0.980, 1.009)	0.988 (0.974, 1.002)	0.986 (0.963, 1.011) *p* = 0.540	0.98 (0.958, 1.004) *p* = 0.140	0.994 (0.970, 1.018) *p* = 1.000
	Ratio of change at 12 months	1.007 (0.993, 1.022)	0.99 (0.974, 1.005)	0.988 (0.973, 1.003)	0.982 (0.957, 1.009) *p* = 0.320	0.98 (0.955, 1.006) *p* = 0.190	0.998 (0.971, 1.024) *p* = 1.000
Insulin^c^ (pmol/L)	Baseline ^d^	(*n* = 131) 51.8 (15.4, 195.7)	(*n* = 124) 56.6 (21.2, 183.9)	(*n* = 134) 53.6 (15.8, 276.8)			
	Ratio of change at 6 months	1.004 (0.953, 1.058)	0.958 (0.908, 1.011)	0.93 (0.883, 0.979)	0.955 (0.871, 1.047) *p* = 0.680	0.926 (0.846, 1.013) *p* = 0.120	0.97 (0.886, 1.062) *p* = 1.000
	Ratio of change at 12 months	1.020 (0.962, 1.082)	0.954 (0.899, 1.014)	0.908 (0.858, 0.962)	0.936 (0.844, 1.037) *p* = 0.370	0.891 (0.806, 0.985) *p* = 0.018	0.952 (0.860, 1.054) *p* = 0.730
HOMA^c^	Baseline ^d^	(*n* = 131) 1.7 (0.5, 8.0)	(*n* = 122) 1.7 (0.4, 6.0)	(*n* = 133) 1.7 (0.6, 8.7)			
	Ratio of change at 6 months	1.011 (0.954, 1.073)	0.956 (0.900, 1.016)	0.918 (0.867, 0.973)	0.946 (0.852, 1.049) *p* = 0.590	0.908 (0.821, 1.004) *p* = 0.066	0.96 (0.866, 1.064) *p* = 1.000
	Ratio of change at 12 months	1.03 (0.965, 1.100)	0.95 (0.888, 1.016)	0.900 (0.844, 0.959)	0.922 (0.822, 1.034) *p* = 0.270	0.873 (0.781, 0.977) *p* = 0.011	0.947 (0.845, 1.062) *p* = 0.760
Total cholesterol (mmol/L)	Baseline	(*n* = 134) 5.3 (1.2)	(*n* = 128) 5.4 (1.2)	(*n* = 135) 5.5 (1.1)			
	Change at 6 months	0.06 (−0.06, 0.18)	−0.13 (−0.25, −0.01)	−0.17 (−0.29, −0.05)	−0.18 (−0.39, 0.02) *p* = 0.1000	−0.23 (−0.43, −0.02) *p* = 0.026	−0.04 (−0.25, 0.17) *p* = 1.000
	Change at 12 months	0.09 (−0.05, 0.23)	−0.10 (−0.24, 0.04)	−0.18 (−0.32, −0.04)	−0.19 (−0.43, 0.05) *p* = 0.180	−0.28 (−0.52, −0.04) *p* = 0.018	−0.08 (−0.33, 0.16) *p* = 1.000
LDL^c^ cholesterol (mmol/ L)	Baseline	(*n* = 134) 3.2 (0.9)	(*n* = 126) 3.3 (0.9)	(*n* = 134) 3.3 (1.0)			
	Change at 6 months	0.02 (−0.12, 0.15)	−0.17 (−0.31, −0.03)	−0.22 (−0.36, −0.09)	−0.19 (−0.42, 0.05) *p* = 0.170	−0.24 (−0.47, −0.01) *p* = 0.040	−0.05 (−0.29, 0.18) *p* = 1.00
	Change at 12 months	−0.02 (−0.16, 0.13)	−0.10 (−0.24, 0.05)	−0.30 (−0.45, −0.16)	−0.08 (−0.33, 0.18) *p* = 1.00	−0.28 (−0.54, −0.04) *p* = 0.020	−0.21 (−0.46, 0.05) *p* = 0.150
HDL cholesterol (mmol/ L)	Baseline ^d^	(*n* = 134) 1.5 (0.7, 3.5)	(*n* = 127) 1.5 (0.8, 3.6)	(*n* = 135) 1.47 (0.8, 3.0)			
	Ratio of change at 6 months	1.019 (0.993, 1.046)	1.019 (0.992, 1.047)	1.013 (0.987, 1.040)	1 (0.955, 1.046) *p* = 1.000	0.994 (0.950, 1.039) *p* = 1.000	0.994 (0.949, 1.040) *p* = 1.000
	Ratio of change at 12 months	1.053 (1.026, 1.082)	1.061 (1.032, 1.090)	1.049 (1.022, 1.077)	1.006 (0.961, 1.053) *p* = 1.000	0.996 (0.951, 1.042) *p* = 1.000	0.99 (0.946, 1.037) *p* = 1.000
Triglycerides^c^ (mmol/ L)	Baseline^d^	(*n* = 134) 1.12 (0.4, 3.3)	(*n* = 127) 1.16 (0.4, 6.5)	(*n* = 135) 1.20 (0.4, 5.8)			
	Ratio of change at 6 months	1.040 (0.985, 1.097)	0.981 (0.929, 1.037)	0.967 (0.916, 1.019)	0.944 (0.859, 1.037) *p* = 0.420	0.930 (0.847, 1.020) *p* = 0.180	0.985 (0.897, 1.082) *p* = 1.000
	Ratio of change at 12 months	1.051 (0.995, 1.111)	0.971 (0.918, 1.027)	0.954 (0.897, 1.008)	0.924 (0.839, 1.107) *p* = 0.150	0.908 (0.826, 0.998) *p* = 0.045	0.982 (0.892, 1.081) *p* = 1.000
Systolic blood pressure (mmHg)	Baseline	(*n* = 137) 124.7 (19.8)	(*n* = 137) 122.4 (20.8)	(*n* = 137) 126.5 (18.5)			
	6 months	1.3 (−1.0, 3.7)	−2.2 (−4.7, 0.2)	−4.1 (−6.2, −2.1)	−4.1 (−7.9, −0.4) *p* = 0.023	−5.0 (−8.7, −1.3) *p* = 0.004	−0.8 (−4.6, 2.9) *p* = 1.000
	12 months	1.1 (−1.5, 3.8)	−1.3 (−3.7, 1.1)	−3.8 (−6.3, −1.3)	−3.1 (−7.1, 0.9) *p* = 0.197	−4.4 (−8.4, −0.4) *p* = 0.025	−1.3 (−5.3, 2.7) *p* = 1.000
12 -min walk test—distance walked (m)	Baseline	(*n* = 125) 927 (208)	(*n* = 127) 901 (218)	(*n* = 128) 928 (190)			
	6 months	59 (39, 79)	74 (52, 95)	108 (86, 130)	15 (−25, 45) *p* = 1.000	49 (15, 84) *p* = 0.002	34 (5, 75) *p* = 0.020
	12 months	92 (69, 11)	103 (78, 127)	131 (105, 156)	11 (−34, 45) *p* = 1.000	39 (0, 79) *p* = 0.050	28 (−5, 73) *p* = 0.115
FACT TOI-F score^e^	Baseline	(*n* = 132) 81.3 (17.1)	(*n* = 128) 78.7 (18.8)	(*n* = 131) 79.3 (18.2)			
	6 months	3.3 (0.5, 6.0)	4.1 (1.6, 6.7)	7.4 (4.4, 10.3)	0.8 (−4.4, 4.3) *p* = 1.000	3.1 (−1.0, 7.7) *p* = 0.194	3.3 (−0.9, 7.8) *p* = 0.176
	12 months	5.1 (2.5, 7.6)	6.7 (4.0, 9.4)	9.4 (6.6, 12.1)	1.6 (−3.5, 4.7) *p* = 1.000	4.3 (−0.5, 7.5) *p* = 0.114	2.7(−1.2, 6.9) *p* = 0.266
FACT TOI-ES score^e^	Baseline	(*n* = 130) 101.7 (15.6)	(*n* = 128) 98.9 (16.5)	(*n* = 130) 100.0 (15.1)			
	6 months	−0.4 (−2.7, 2.0)	2.6 (0.6, 4.6)	3.6 (1.1, 6.0)	3.0 (−1.5, 5.9) *p* = 0.460	4.0 (−0.2, 7.1) *p* = 0.069	1.0 (−2.4, 5.0) *p* = 1.000
	12 months	0.9 (−1.4, 3.2)	3.1 (0.9, 5.1)	4.5 (2.1, 6.7)	2.2 (−2.3, 4.9) *p* = 1.000	3.6 (−0.6, 6.6) *p* = 0.139	1.4 (−1.9, 5.3) *p* = 0.799
FACT TOI-BC score^e^	Baseline	(*n* = 130) 66.0 (13.3)	(*n* = 127) 64.1 (13.7)	(*n* = 130) 65.3 (12.9)			
	6 months	2.9 (1.0, 4.7)	4.0 (2.3, 5.6)	6.2 (4.2, 8.1)	1.1 (−2.3, 3.5) *p* = 1.000	3.3 (0.2, 6.0) *p* = 0.029	2.2 (−0.39, 5.4) *p* = 0.113
	12 months	4.7 (2.9, 6.5)	6.4 (4.5, 8.3)	8.3 (6.6, 10.0)	1.7 (−1.7,3.8) *p* = 1.000	3.6 (0.6, 6.1) *p* = 0.011	1.9 (−1.8, 6.4) *p* = 0.138

Mean (SD)

^a^ ANCOVA, Mean (95% CI)

^b^ ANCOVA with Bonferroni adjustment, mean (95% CI)

^c^ Patients with fasting baseline values were included into summaries and analyses

^d^ Geometric mean (range)

^e^ FACT analysis: TOI  =  Trial outcome indicator for fatigue  =  PWB + FWB + FSS

TOI-ES  =  Trial outcome indicator for endocrine system  =  PWB + FWB + ESS

TOI-BC  =  Trial outcome indicator for breast cancer  =  PWB + FWB + BC

*Quality of life*: There were numerical improvements in the scores for QOL (FACT TOI for fatigue, endocrine symptoms and BC) in all three groups at 6 and 12 months. The improvement reached significance using the FACT TOI BC at 6 and 12 months for the community group compared with controls (both *p* < 0.05) but not the home group compared with controls (Table 4).

Changes in dietary intake and physical activity: All three groups reduced energy intake (Supplementary Table 2). The greatest numerical reductions were reported by the home group; mean (95% CI) difference in the home vs. control group was −153 (−239, −68) kcal/day (*P* = 0.001) at 6 months and −161 (−261, −61) kcal/day at 12 months (*p* < 0.001), whilst mean (95% CI) difference in energy intake in the community vs. control group was −82 (−185, + 20) kcal/day at 6 months (*P* = 0.166) and −107 (−205, −9) kcal/day at 12 months (*P* = 0.027). Reported levels of PA increased in all three groups over the year (Supplementary Table 2). At 12 months the community group had significantly greater increases compared to the control and home groups; mean (95% CI) difference community vs. control 119 (6, 230) minutes/week (*P* = 0.035) and mean (95% CI) difference community vs. home group 151 (36 to 265) minutes/week (*P* = 0.005).

#### **Economic evaluation**

Disaggregated and mean total health care costs and mean QALYs of the three interventions are reported in Supplementary Tables 3 and 4. The home group (£7737) and the community group (£7914) had reduced patient costs compared with the control group (£8547) mainly related to decreased usage of medications to treat treatment toxicity, e.g. granulocyte-colony stimulating factor (GCSF) used in chemotherapy patients who had become neutropenic, decreased accident and emergency visits and physiotherapy contacts. The three interventions had equivalent QALY scores of ~0.8. Comparing the difference in costs and difference in effects between the home and community groups gives an incremental cost-effectiveness ratio (ICER) of £9381.45. For a threshold of £20,000 per QALY there is a 52% probability that the community group is cost-effective; this increases to 60% for a £30,000 per QALY threshold (Supplementary Fig. 1).

## Discussion

The three-month dietary and PA weight control programmes initiated soon after surgery produced sustained weight reduction in overweight women and prevented weight gain in normal-weight women during the 12 months of the study. Although equally effective for weight control, the community programme was more effective for increasing PA, whilst the home programme appeared more effective for dietary change and reducing energy intake. The greater PA in the community group probably accounts for the greater reductions in insulin, lipids^33^ and blood pressure,^34^ and improvements in fitness and QOL.^35^ Neither programme was effective for inducing weight loss or preventing weight gain amongst patients receiving chemotherapy.

Long term behaviour change is pivotal to the effectiveness of any weight control programme. Beneficial changes in diet and PA behaviours, weight, CVD disease biomarkers, and QOL were observed at 12 months, i.e. 9 months after completion of the initial 3-month phone and community programmes. Some evidence suggests that uptake of interventions and maintenance of behaviours within studies are greater when were commenced nearer to the time of diagnosis compared with those initiated later although there are no randomised trials comparing early versus later interventions.^36^ Few studies have assessed the maintenance of diet and PA behaviour change and weight loss beyond the end of the intervention.^36^ The respective maintained and continued weight loss in the home and supervised programmes between 6 and 12 months (i.e. 3–9 months after the intensive 12-week intervention) is an important finding. Both groups did receive minimal ongoing contact via booster calls at 4 and 9 months. This ongoing albeit minimal contact is likely to have contributed to their maintained lifestyle behaviour change. Average weight loss in the overweight/obese non-chemotherapy patients was 3 kg (4%) when assessed 9 months after the end of the active intervention. This compares favourably with reported 12-month weight loss at the end of a 12-month active intervention amongst women who joined the programmes 9–60 months after diagnosis summarised by Chlebowski and Reeves et al. which ranged from 3.7–5 kg (4–5%).^10^ This level of weight loss may be clinically important for BC patients. A large randomised trial of low-fat dietary intervention (Women’s Intervention Nutrition Study, WINS)^37^ reported a 24% reduction in relapse where women lost on average 2.3 kg (3%).

Home based home diet and PA programmes have been shown to be equivalent to face to face versions for weight loss amongst BC patients after adjuvant treatment,^38^ and amongst other patient groups.^39,40^ Home based mail and web programmes are effective for changing diet and weight, but they only have limited effects on PA as reported here.^41^ PA increases more within supervised programmes^42^ as these overcome common barriers including women’s concerns about safety and low self-efficacy for physical activity.^43^ The independent effects of PA on toxicity^44^ and possibly BC outcome^45^ mean that programmes need to impact on PA as well as diet and weight. Future trials should test home based programmes which include initial supervised PA sessions and more intensive self-monitoring and feedback, which have proven to successfully promote PA in home based CVD rehabilitation and heart failure programmes.^46,47^

Weight gain was observed in the normal weight but not the overweight controls which is consistent with previous reports in the literature.^18^ The weight control advice was effective for preventing these gains amongst patients who were not receiving chemotherapy. It is important to note that none of the normal-weight patients reduced weight to a BMI of < 18.5 kg/m^2^ and so did not require weight gain advice.

Chemotherapy patients were keen to enter the programmes, and were equally likely to attend the community sessions or receive calls, but slightly more likely to drop out. Both programmes appeared to confer some benefits to the chemotherapy patients in terms of reduced costs of toxicity related medication (e.g. GCSF) and accident and emergency admissions, but they were ineffective for weight control during chemotherapy and in the post-treatment phase up to 12 months. The limited success of home^12,13^ and group^48^ weight control programmes during adjuvant chemotherapy has been reported previously in BC patients, with the exception of an intensive dietary intervention which involved twice-weekly community cookery classes and group meals.^49^ However, Goodwin et al. reported that women who had previously received chemotherapy achieved comparable successful weight loss to women who had not previously received chemotherapy in a home-based phone weight loss programme initiated at a median of 9 months from diagnosis and at least 1 month after completion of adjuvant chemotherapy.^50^ Future studies should test modified, more achievable approaches amongst chemotherapy patients, e.g. intermittent energy restriction which is effective for weight loss in the non-cancer setting.^51^

The combined diet and PA community programme was the most cost-effective. There are few data on the cost-effectiveness of programmes amongst early BC patients.^52^ Two earlier studies failed to demonstrate the cost-effectiveness of PA only programmes during adjuvant treatments which were home^53^ or community^54^ based.

The strengths of this study include random allocation to the three groups, DXA assessment of body composition, and 12 months follow up. We have previously reported the good uptake to the trial and that our cohort is representative of newly diagnosed early-stage BC patients,^32^ whilst the low drop-out provides reliable follow up data without making assumptions about missing data. We tested 12-week programmes. The optimal length of programme for sustained behaviour change for weight loss is not known. Some guidelines advocate a minimum of 16 contacts over a 6-month period as used in the Diabetes Prevention Programme,^55^ whilst others advocate a minimum of 12 weeks.^56^ It is estimated to take 10 weeks to form a habit.^57^ Both of our groups involved weekly contacts. However, half of the contacts in the home-based group were mailings rather than direct patient contact, hence this group had reduced contact with their allocated dietitian which may have limited the effectiveness of the programme. The reduced effects on physical activity in the home-based group is likely to reflect the home-based modality rather than the level of contact. Poor collective results of home-based PA interventions have been reported amongst patients with BC regardless of the length of intensity of the intervention.^58^

Limitations include that the sample size may not be sufficiently powered for the subgroup analyses in chemotherapy and non-chemotherapy patients and the use of self-report rather than objective measurements of PA such as accelerometry. Our metrics for assessing adherence were based on retention to the study and attendance to classes and receipt of the calls. Future studies should evaluate more detailed adherence to the diet and PA prescriptions which would give a more meaningful evaluation of engagement with the programmes.

We have shown significant numbers of BC patients are interested and motivated to enter and adhere to home and community-based diet and PA weight control programmes soon after diagnosis. Lifestyle programmes in current oncology practice are mainly focussed at the end of active treatment.^59,60^ This has been identified as a time of need amongst patients,^61^ but it means that programmes are initiated after women may have already gained weight as a result of the psychological and physical effects of BC diagnosis and treatment. Research should focus on developing cost-effective interventions for women soon after diagnosis to utilise this potential teachable moment. Such programmes are likely to improve the future health of women affected by BC by reducing future weight-related illness and improving QOL. Ongoing randomised trials^62^ will inform the potential effectiveness of weight control programmes for improving BC specific outcomes for BC patients long after diagnosis and treatment.

## Supplementary information

Supplementary information summary

Supplementary Figure 1

Supplementary Table 1

Supplementary Table 2

Supplementary Table 3

Supplementary Table 4

## Data Availability

All datasets used and analysed during the current study are available from the corresponding author on reasonable request.
