# Supplementary material for: The effectiveness of home versus community-based weight control programmes initiated soon after breast cancer diagnosis: a randomised controlled trial
Source: Br J Cancer. 2019 Aug 1;121(6):443–54. doi: 10.1038/s41416-019-0522-6 (PMC6738088; doi:10.1038/s41416-019-0522-6)
Supplement: Supplementary file 1 — Supplementary information summary [file 41416_2019_522_MOESM1_ESM.docx]

#### Supplementary information summary

Table 1: Twelve weekly topics covered in education sessions in the community group and mailings for the home group.

#### Table 2: Changes in self-reported diet and physical activity over 12 months

#### Table 3: Mean cost per patient of all outpatient, primary care and community care contacts, hospital inpatient admissions, and Quality Adjusted Life Years to 12 months for each of the three trial interventions over 12 months

#### Table 4: Breakdown of the mean total costs per patient of each of the three trial interventions over 12 months

Figure 1: Cost-effectiveness acceptability curves showing the probability that each trial intervention is cost-effective at different cost-effectiveness threshold values
