# Supplementary figures and images for: The effectiveness of home versus community-based weight control programmes initiated soon after breast cancer diagnosis: a randomised controlled trial
Source: Br J Cancer. 2019 Aug 1;121(6):443–54. doi: 10.1038/s41416-019-0522-6 (PMC6738088; doi:10.1038/s41416-019-0522-6)

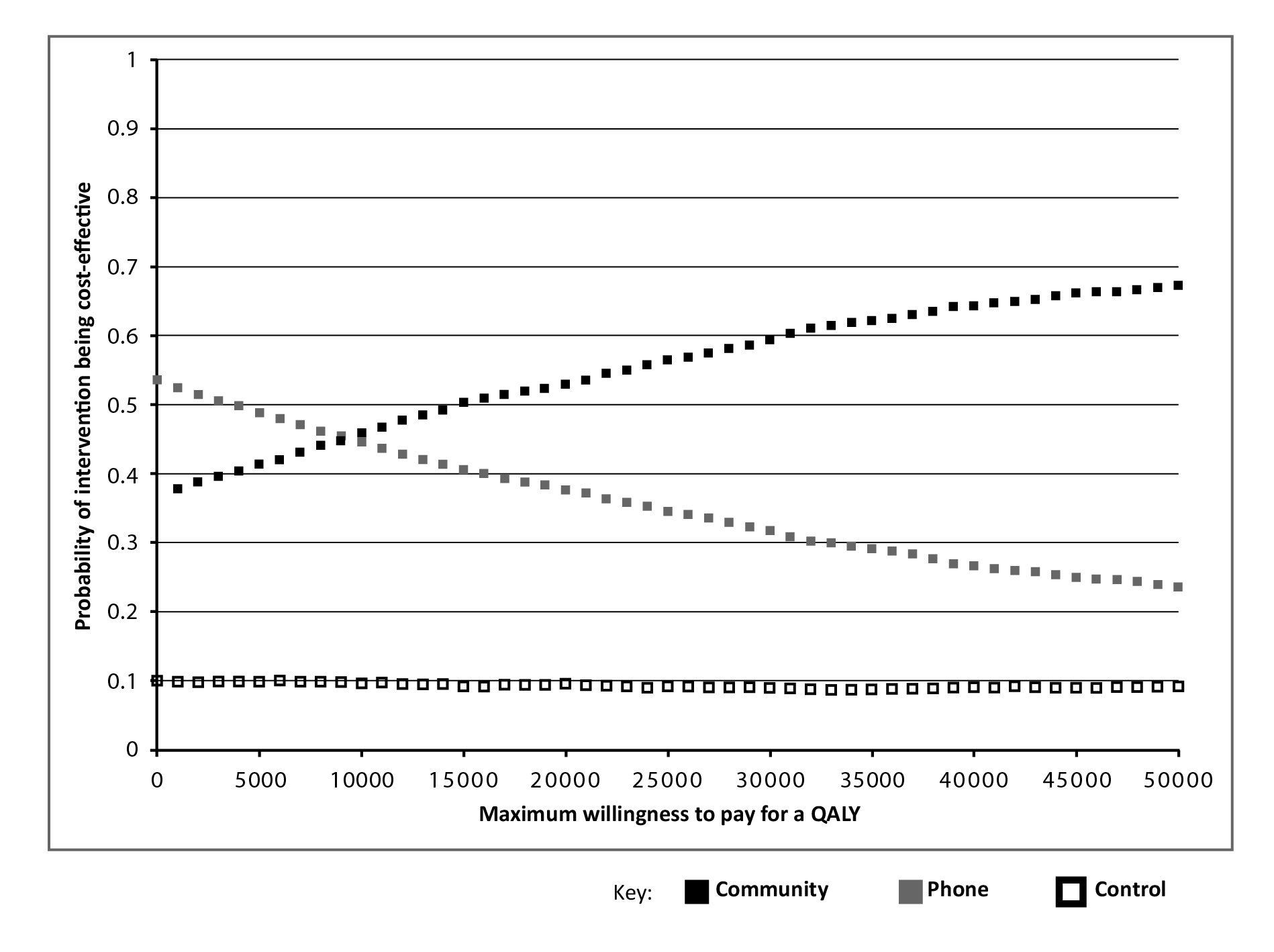

Supplement: Supplementary file 2 — Supplementary Figure 1 [file 41416_2019_522_MOESM2_ESM.jpg]
